# Model-Based Virtual Clinical Trial Reveals Renal Impairment and Body Size as Key Determinants of Pharmacokinetic Variability and Drug-Drug Interaction Risk in Propranolol Therapy

**DOI:** 10.3390/pharmaceutics18060636

**Published:** 2026-05-22

**Authors:** Lara Marques, Nuno Vale

**Affiliations:** 1 PerMed Research Group, RISE-Health, Faculty of Medicine, University of Porto, Alameda Professor Hernâni Monteiro, 4200-319 Porto, Portugal; lfmarques@med.up.pt; 2 RISE-Health, Department of Community Medicine, Health Information and Decision (MEDCIDS), Faculty of Medicine, University of Porto, Rua Doutor Plácido da Costa, 4200-450 Porto, Portugal; 3 Laboratory of Personalized Medicine, Department of Community Medicine, Health Information and Decision (MEDCIDS), Faculty of Medicine, University of Porto, Rua Doutor Plácido da Costa, 4200-450 Porto, Portugal

**Keywords:** propranolol, omeprazole, popPK modeling, PBPK modeling, pharmacokinetics, drug-drug interactions, covariates, interindividual variability, personalized medicine

## Abstract

**Background/Objectives:** Propranolol (PROP) is a non-selective β-blocker widely prescribed for cardiovascular and neurological disorders. Its pharmacokinetics (PK) are highly variable, and co-administration with omeprazole (OME), a CYP2C19 substrate and inhibitor, may alter systemic exposure. Herein, this study aimed to investigate factors influencing PROP PK variability and evaluate the effect of OME coadministration using physiologically based pharmacokinetic (PBPK) modeling and population PK (popPK) analysis. **Methods:** PBPK models for PROP and OME were developed and validated against published data. DDI simulations were conducted across clinically relevant dosing regimens. A two-period fixed-sequence virtual trial of 125 subjects was simulated with PROP alone and PROP combined with OME. Population PK (popPK) analysis was performed on simulated plasma concentration data to identify covariates affecting PROP disposition and quantify DDI magnitude. **Results:** PBPK models were successfully developed and validated. PROP disposition was best described by a two-compartment model with linear elimination. Health status was found to influence clearance, and body surface area (BSA) affected the central volume of distribution. Co-administration with OME increased PROP exposure, with larger effects in patients with renal impairment. Simulated plasma concentrations remained below established toxicity thresholds. **Conclusions:** Virtual clinical trials integrating PBPK and popPK modeling provide a robust approach to identifying key determinants of PK variability and DDI risk. Although these findings were not directly translated to clinical observations, this helps identify sources of PK variability in PROP treatment settings and factors that may intensify its interaction with OME, thereby supporting model-informed precision dosing to enhance safety and efficacy.

## 1. Introduction

Propranolol (PROP) is a non-selective β-adrenergic receptor antagonist belonging to the β-blocker class [1,2,3,4]. It is indicated for the management of cardiovascular diseases (CVD), including arrhythmias, ischemic heart disease, heart failure, and hypertension, as well as non-cardiovascular conditions such as migraine, anxiety disorders, thyrotoxicosis, essential tremors, hyperthyroidism, and infantile hemangiomas [5,6]. CVD remains the leading cause of mortality worldwide [7], accounting for an estimated 19.8 million deaths in 2022, which represents approximately 32% of all global deaths [8]. In this context, the clinical use of β-blockers, particularly PROP, remains of major therapeutic importance [9,10]. In addition to its widespread cardiovascular indications, PROP is extensively prescribed for neurological and central nervous system-related disorders [3]. Consequently, it is among the drugs most frequently implicated in both intentional and unintentional poisonings. Epidemiological studies consistently report its involvement in severe and fatal outcomes [3]. For instance, a study conducted in Iran involving 255 overdose cases identified PROP as the causative agent in approximately 84% of cases, predominantly affecting young women with underlying psychiatric disorders and prior suicide attempts [11].

These observations are largely attributable to the unique pharmacological properties of PROP. Following oral administration, PROP is rapidly absorbed and undergoes extensive hepatic first-pass metabolism, resulting in low systemic bioavailability [12,13]. Owing to its high lipophilicity, it is widely distributed throughout body tissues and exhibits a high affinity for plasma protein binding [12,13,14,15]. Elimination occurs predominantly via hepatic metabolism, with only approximately 1–4% of the administered dose recovered as unchanged drug in urine and feces [12,13,16,17]. The principal metabolic pathways of PROP include: (i) aromatic ring hydroxylation at the 4-position, (ii) side-chain N-desisopropylation, and (iii) direct glucuronidation, leading to the formation of active metabolites such as 4-hydroxypropranolol [13].

Although the pharmacokinetics (PK) of PROP have been extensively characterized and may appear straightforward at first glance, its oral disposition is inherently complex. PROP exhibits nonlinear PK, primarily due to saturation of high-affinity hepatic uptake processes or binding sites, resulting from elevated drug concentrations in the portal vein following oral administration [13,18]. Saturation of metabolic pathways has been observed and is consistent with the formation of the active metabolite 4-hydroxypropranolol predominantly after oral dosing [16]. At lower doses, systemic bioavailability is relatively lower compared with higher doses [19], supporting the presence of dose-dependent presystemic extraction. Prolonged treatment further highlights this saturable behavior, with pre-systemic extraction decreasing from approximately 78% after the first dose to 66% at steady state, suggesting partial saturation of drug-metabolizing enzymes [14,20]. In vitro investigations evaluating the role of cytochrome P450 (CYP) enzymes in PROP metabolism have demonstrated that CYP1A2 is the primary enzyme responsible for N-desisopropylation [21]. CYP2C19 contributes to both N-desisoproylation and oxidative deamination of N-desisopropyl-propranolol, whereas CYP2D6 predominantly mediates aromatic ring hydroxylation.

These PK properties, particularly its high lipophilicity and its central nervous system (CNS) penetration, are closely associated with PROP-related toxicity [3,12,13,17]. Indeed, cases of PROP poisoning are reported annually, not only due to its high prescription rates but also because of its broad therapeutic indications, including anxiety and depressive disorders. PROP accounts for the majority (84.4%) of β-blocker toxicity cases [11]. Data from the UK Poison Centre indicate 363 intentional PROP overdoses involving 359 patients, with PROP having been prescribed for anxiety in 111 of these individuals [22]. Furthermore, in 43 out of 61 cases where PROP was coadministered with an antidepressant, both agents were ingested in overdose. The most frequently reported adverse drug reactions (ADRs) include neurological manifestations such as seizures and coma, due to the compound’s ability to cross the blood–brain barrier (BBB) [3,23]. In addition, its non-selective β1/β2-adrenergic receptor blockade precipitates bradycardia, hypotension, bronchospasm, and hypoglycemia, all of which may contribute to severe or fatal outcomes in overdose scenarios. Given the high incidence of serious adverse events associated with PROP, careful and individualized prescribing is warranted. This requires a thorough evaluation of patient-specific factors that may influence PK, pharmacodynamics (PD), and overall toxicity risk.

Managing interindividual variability (IIV) in PK and PD parameters remains a major challenge in achieving therapeutic efficacy and drug safety across diverse patient populations in the era of personalized medicine. Variability in patient response to drug therapy is well documented and arises from multiple contributing factors, including genetic makeup, disease states, age, sex, drug-drug interactions (DDIs), and environmental influences such as diet, alcohol consumption, and smoking [23]. This variability may lead to therapeutic failure when plasma concentrations do not reach the predefined efficacy threshold, or to toxicity when concentrations exceed established safety margins. Among these determinants, DDIs assume particular importance in a context of increasing multimorbidity—defined as the coexistence of two or more chronic diseases in the same individual—which consequently drives polypharmacy, i.e., the concurrent use of multiple medications. Evidence suggests that the prevalence of polypharmacy ranges between 20% and 60% [24]. A meta-analysis conducted by Aksoy & Ozturk [25] investigating the prevalence of DDIs among hospitalized patients demonstrated a significant increase in both clinically manifest and potential DDIs.

Several PK interactions involving PROP have been reported: phenytoin, rifampicin, chlorpromazine, cimetidine, etc. [19]. The most clinically relevant PK interactions typically occur at the level of CYP enzymes, which may be induced or inhibited, thereby altering the clearance (C*l*) of co-administered drugs. One drug with potential metabolic interaction with PROP at the CYP level is omeprazole (OME). OME is a proton pump inhibitor that irreversibly inhibits the gastric H^+^/K^+^-ATPase in parietal cells, leading to suppression of gastric acid secretion. It is widely prescribed for the treatment of gastrointestinal (GI) disorders, including gastroesophageal reflux disease (GERD), peptic ulcers, and *Helicobacter pylori* infection. OME is primarily metabolized by CYP2C19 and CYP3A4 and is also a potent inhibitor of these enzymes [26]. Therefore, PROP and OME share CYP2C19 as a common metabolic pathway, providing a mechanistic basis for a potential metabolic interaction. In a randomized, double-blind, crossover study investigating the DDI between PROP and OME, eight healthy individuals received PROP 80 mg twice daily in combination with OME 20 mg or placebo [27]. The findings suggest that OME at therapeutic doses typically used for peptic ulcer disease did not significantly affect the PK of PROP. However, data regarding other combined therapeutic dosing regimens remain scarce, and such regimens may result in clinically relevant interactions.

Importantly, the high PK variability reported for PROP may further amplify any inhibitory or competitive effects exerted by OME. Ågesen et al. [28] evaluated the PK variability of PROP in healthy individuals receiving single or multiple doses and identified 40 single-dose studies and 36 steady-state studies reporting a coefficient of variation (CV) greater than 40% for area under the curve (AUC), classifying PROP as a highly variable drug. Moreover, plasma concentrations of PROP exhibit substantially greater variability following single oral doses (4- to 20-fold range) [13,29], reinforcing the contribution of presystemic processes to variability.

There has been growing recognition of the need to incorporate model-informed precision dosing (MIPD) and model-informed drug development (MIDD) strategies to address diverse PK/PD challenges. Recently, virtual patients and in silico clinical trials (also referred to as virtual clinical trials) have emerged as innovative approaches capable of overcoming many of the operational, ethical, and logistical constraints associated with conventional clinical trials [30,31,32]. Virtual patients are computer-generated simulations based on mathematical modeling that replicate the clinical characteristics of real individuals, enabling researchers to simulate clinical trials without involving human participants [30]. This approach is particularly valuable for identifying IIV in the PK of approved and marketed drugs, thereby supporting the optimization of efficacy and safety during the critical pharmacovigilance phase. A more audacious concept involves virtual twins (VTs) integrated with physiologically based pharmacokinetic (PBPK) modeling [33,34,35]. This framework incorporates real patient demographic, biochemical, and physiological data to generate patient-specific digital counterparts, enabling physiologically realistic modeling of drug disposition. Although still limited in widespread application, VT-PBPK modeling shows strong promise for characterizing IIV in PK and improving individualized predictions [35,36,37,38]. Nevertheless, the present study does not yet constitute a VT-PBPK model.

In this context, this study aimed to identify sources of IIV in the co-administration of PROP and OME using a model-based virtual clinical trial framework. A fixed-sequence, two-period virtual clinical study was designed in which virtual patients received PROP alone during the first period, followed by a washout phase, and subsequently received PROP in combination with OME during the second period. Predictions were performed using PBPK modeling, both for virtual population generation and for the simulation of individual concentration-time profiles. These simulated data were subsequently analyzed using population pharmacokinetic (popPK) modeling to identify covariates significantly influencing PROP PK parameters. Secondarily, the magnitude of the DDI between PROP and OME was evaluated across multiple therapeutic scenarios, and patient-specific factors potentially exacerbating this interaction were systematically investigated.

## 2. Materials and Methods

### 2.1. Software

The software GastroPlus^®^ (version 9.9.; Simulation Plus Inc., Lancaster, CA, USA) was used for the development and verification of the PBPK models for OME and PROP. These models were constructed using parameter values extracted from the literature; when such data were unavailable in published studies, parameters were estimated and optimized using ADMET Predictor^®^ (version 12.0; Simulation Plus Inc., Lancaster, CA, USA) to derive physicochemical and PK properties from the chemical structures of OME and PROP, which were generated using MedChem Designer^®^ (Version 10.0, Simulation Plus Inc., Lancaster, CA, USA). Concentration-time profiles collected from the literature were digitized using WebPlot Digitizer (Version 3.4; https://web.eecs.utk.edu) (accessed on 13 January 2026). OpenAI’s ChatGPT (Version GPT-4, OpenAI, San Francisco, CA, USA) was also used to assist in refining digitized clinical plasma data by incorporating reported maximum plasma concentration (C_max_) and time to reach maximum plasma concentration (T_max_) values to adjust the extracted concentration–time profiles and improve alignment with the original published PK parameters. Data management, visualization, and analysis were performed using GraphPad Prism (Version 10.6.1, GraphPad Software, Boston, MA, USA). The popPK analysis was conducted using a nonlinear mixed-effects (NLME) approach in Monolix (Version 2024R1, Simulation Plus Inc., Lancaster, CA, USA), and the data were initially evaluated by noncompartmental analysis (NCA) using PKAnalix (Version 2024R1, Simulation Plus Inc., Lancaster, CA, USA).

### 2.2. Stepwise PBPK Model Construction

#### 2.2.1. Model Development of Propranolol

The PBPK model for PROP was developed using the demo model available in GastroPlus^®^ as a structural framework. Drug-specific parameters for PROP are detailed in Table 1. Some input values were optimized based on the PBPK model reported by Kalam et al. [1], particularly those related to drug distribution and metabolism. The PBPK model comprised 13 compartments, including the lungs, adipose, muscle, liver, spleen, heart, brain, kidneys, skin, reproductive organs, red marrow, yellow marrow, and the rest of the body. Each compartment was characterized by its volume, an associated tissue blood flow rate, and tissue-to-plasma partition coefficients (Kp). The Poulin and Theil Extracellular method [39] was used for Kp estimation, and perfusion rate-limited distribution was assumed for all tissues. Oral absorption was simulated using the Advanced Compartmental Absorption and Transit (ACAT) model under human physiological fasted conditions, as implemented by default in GastroPlus.

#### 2.2.2. Model Development of Omeprazole

A whole-body PBPK model for OME and its major metabolites, hydroxy-omeprazole (hydroxy-OME) and OME sulphone, was developed and evaluated following the same approach described by Soliman et al. [40]. Drug-specific parameters incorporated into the model were extracted from the study by Soliman et al. [40], which integrates data from a comprehensive literature review combining physicochemical and biopharmaceutical parameters with clinical and PK information, as well as parameters predicted using ADMET Predictor^®^. The dissolution model (z factor) was implemented in accordance with this study.

Similarly, the drug metabolism information incorporated into the PBPK model was initially based on the same work [40] and subsequently manually optimized to improve the fit of the PK profiles to the clinical data used for model development. The main metabolites of OME include hydroxy-OME, 5′-O-desmethylomeprazole, OME sulphone, and carboxy-omeprazole (carboxy-OME) [41,42]. To achieve a comprehensive PBPK model, the contributions of CYP2C19, CYP2C9, and CYP3A4 to the formation of hydroxy-OME and OME sulphone, as well as their subsequent metabolism, were incorporated [43,44]. The data relevant to the construction of the PBPK models for the metabolites hydroxy-OME and OME sulphone are presented in Table S1, Supplementary Material. Accordingly, the metabolic processes included in the model were: (i) hepatic metabolism of OME by CYP2C19 to form hydroxy-OME and other metabolites, including 5′-O-desmethylomeprazole; (ii) hepatic metabolism of OME by CYP3A4 to form hydroxy-OME, OME sulphone, and other metabolites such as 5′-O-desmethylomeprazole; (iii) intestinal metabolism of OME by CYP3A4 to form OME sulphone; and (iv) hepatic metabolism of OME by CYP2C9 to form hydroxy-OME and other metabolites such as 5′-O-desmethylomeprazole. Metabolism by these CYP enzymes was parameterized using reported Michaelis-Menten constant (Km) and maximum reaction velocity (Vmax) values, which were subsequently adjusted to fit the PBPK model to the observed plasma data. Enzyme expression levels were set to the default values implemented in GastroPlus^®^. All input data are summarized in Table 2.

The intestine was modeled using the ACAT model built into GastroPlus^®^, with the addition of a zero-order gastric emptying process to improve model fitting. This assumption implies that gastric contents are transferred to the gut at a constant rate over time, independent of the remaining amount in the stomach. Consistent with Soliman et al. [40], Kp for perfusion-limited tissues (liver, lung, spleen, adipose, heart, kidney, reproductive organs, yellow marrow, muscle, brain, skin, red marrow, and the rest of the body) were calculated using the Lukacova method [50].

To simulate plasma concentration-time profiles, the clinical study conducted by Andersson et al. [41] was used for both the development and subsequent validation of the PBPK model. The same population characteristics reported in the clinical study were simulated, and the mean population profile was generated. Human organ weights, volumes, and blood perfusion rates specific to subjects in each study (sex, age, and body weight) were generated using the GastroPlus^®^ internal Population Estimates for Age-Related Physiology (PEAR) Module. Incorporation of metabolites into the OME PBPK model additionally enabled the prediction of the PK profiles of these metabolites.

### 2.3. PBPK Model Evaluation and Validation of Propranolol and Omeprazole

The developed PBPK models were evaluated through visual inspection of the PK profile and by comparison of several PK parameters with previously reported clinical data. Clinical plasma concentration-time data following single-dose administration of OME and PROP were collected and digitized from the literature. In total, four clinical studies comprising 54 subjects were used for model development and validation. A description of these studies is summarized in Table 3.

For development and validation of the PROP PBPK model, a single oral (tablet) dose of 5 mg PROP was simulated in a healthy 30-year-old American male subject weighing 75 kg, and the simulated PK profile was visually compared with the data reported by Mould et al., 1981 [53]. The model was then applied to simulate PK profiles following single oral doses of 10, 40, and 80 mg PROP. These predictions were evaluated against clinical data reported by Wang et al., 2013 [54], Mould et al., 1981 [53], and Giudicelli et al., 1977 [55], respectively.

The PBPK model for OME was developed based on the clinical study conducted by Andersson et al., 1990 [41], for which a full OME PBPK model incorporating its major metabolites was established. The same population characteristics reported in this study were simulated, and the mean population profile was generated. For model building, plasma concentration-time data following single intravenous (IV) doses of 40 mg and 10 mg OME were used. Subsequently, PK profiles following single oral doses of 40 mg and 90 mg OME reported by the same authors were simulated.

The arithmetic mean concentration-time profiles were compared with the mean concentration-time curves reported in the corresponding clinical studies. In addition, ratios between observed and predicted PK parameters were determined. A two-fold error was considered acceptable for model validation, as previously reported [56,57]. Furthermore, statistical metrics, including the average fold error (AFE) and the root mean square error (RMSE), were calculated for further validation of the developed PBPK model. AFE was used to assess systemic bias, whereas RMSE quantified the impact of larger deviations between observed and predicted values, thereby enhancing the overall robustness of the model validation process [58,59].

### 2.4. Quantitative Prediction of DDI

To quantitatively and qualitatively investigate the potential interaction of OME coadministration on the PK of PROP, the GastroPlus^®^ DDI module was used in both steady-state and dynamic simulation modes. OME was defined as the perpetrator drug, and PROP as the victim drug. Table 4 summarizes the input parameters used for the DDI simulations, including the inhibition constant (K_i_) and the enzyme inactivation rate constant (K_inact_) of OME toward CYP2C19 and CYP3A4, obtained from the literature. It also includes the metabolic profile detected from the PROP PBPK model. Although PROP is primarily metabolized by CYP1A2, CYP2C19, and CYP2D6, CYP3A4 was also incorporated into the DDI assessment, as it contributes to the secondary metabolic pathway of this compound.

The simulations were conducted according to the previously developed PBPK models. Initially, the interaction between OME and PROP was predicted using a standard PBPK model representing a 30-year-old American male under steady-state conditions. Four PROP dosing regimens were combined with four OME dosing regimens: PROP was simulated at 40 mg twice daily (q12h), 80 mg once daily (OD), 10 mg every 6 h (q6h), and 160 mg OD, while OME was simulated at 20 mg OD, 40 mg q12h, 40 mg OD, and 60 mg OD. These therapeutic regimens were selected based on the most commonly reported dosing schemes in the literature [62,63]. DDI classification was based on the AUC ratio in the presence versus absence of the perpetrator drug. Interactions are categorized as no interaction, weak (AUC ratio 1.25–2), moderate (AUC ratio 2–5), or strong (AUC ratio > 5) [64,65].

Subsequently, the highest OME dose (60 mg OD) and the most common and clinically relevant PROP dose (40 mg q12h) were selected to represent a clinically plausible worst-case exposure scenario. This approach enabled the evaluation of dynamic DDI effects across different patient populations and allowed the subsequent identification of patient-specific covariates that may exacerbate the interaction. It was assumed that the software automatically adjusted physiological input parameters to account for the intergroup physiological differences among the simulated patient populations.

### 2.5. Virtual Clinical Trial

A total of 125 virtual patients were generated using the internal GastroPlus PEAR Physiology^TM^ module, which predicts organ physiology parameters for American, Japanese, and Chinese human populations across a wide age range, from premature neonates to 85 years old. The inclusion criteria applied in this study were: age 18–80 years, body mass index (BMI) between 15.64 and 35 kg/m^2^, and body weight between 54 and 115 kg. The generated virtual population was stratified into five distinct groups according to ethnicity or health status (Table 5). The male–female proportion was 50%.

To simulate a fixed-sequence, two-period clinical trial—the most common clinical DDI study design, in which the victim drug is administered alone and subsequently in combination with the inhibitor—virtual subjects first received 40 mg q12h of PROP as monotherapy during the first period. Following a washout phase, they received 40 mg q12h of PROP coadministered with 60 mg OD of OME during the second period. The PROP dose of 40 mg q12h was selected as it represents a clinically relevant and commonly prescribed regimen. Although 60 mg OD is not the standard OME dose, it is used in specific clinical scenarios such as refractory gastroesophageal reflux disease (GERD), severe erosive esophagitis, and gastrointestinal bleeding prophylaxis in high-risk patients [63]. The combination of these dosing regimens was intended to represent a clinically plausible high-exposure scenario.

Drug disposition-related parameters were simulated over a 24 h period for each patient group, with concentration–time data collected at 6 min intervals. As a result of these simulations, output datasets were generated containing comprehensive physiological characteristics of each virtual subject—age, weight, height, body surface area (BSA), gender, ethnicity, health status (healthy or renally impaired), and coadministration with OME—as well as the corresponding plasma concentration-time profiles following PROP monotherapy and PROP coadministered with OME. This study design enabled direct comparison of the PROP PK profile within each individual under both treatment conditions.

### 2.6. Population Pharmacokinetic Modeling

A dataset containing demographic information and plasma concentration–time data points of 125 virtual patients (30,250 observations) was prepared for integration into the population analysis to model PROP under both monotherapy and coadministration with OME within the same individual. This approach enabled the estimation of IIV and the quantification of OME as a covariate effect. To distinguish between the PROP PK profiles under monotherapy and coadministration conditions, time was reset, and an occasion variable was introduced. Occasion coded as 1 corresponded to PROP alone, whereas occasion coded as 2 corresponded to PROP + OME.

A preliminary data analysis was conducted using noncompartmental analysis (NCA) in PKAnalix (Version 2024R1, Lixoft, Antony, France) to characterize the drug disposition profile. The linear trapezoidal method with equal weights assigned to each data point was applied to calculate AUC. Selection of data points for the terminal elimination phase was guided by the adjusted R^2^ criterion (maximized toward 1), enabling the estimation of the terminal slope and calculation of the elimination rate constant (λ_z_).

The Monolix software (Version 2024R1, Lixoft, Antony, France) was used to perform the popPK analysis using a nonlinear mixed-effects (NLME) modeling approach. PK parameters were estimated by maximum likelihood using the Stochastic Approximation Expectation-Maximization (SAEM) algorithm. The base structural model was developed using an empirical approach. One-, two-, and three-compartment models were evaluated for extravascular administration of PROP, incorporating either first-order or zero-order absorption, with or without lag time, and linear or Michaelis-Menten elimination (Table S2). Exploratory data analysis and prior knowledge of the typical PK of PROP informed the selection of the structural model functional form. PK parameters were initially assumed to follow a normal distribution. A proportional error model was applied to describe residual variability. Initial parameter estimates were automatically computed by the software using all individuals included in the study, selecting population parameter values that best fit the observed data. The most appropriate structural model was systematically selected based on the lowest corrected Bayesian Information Criterion (BICc), alongside visual inspection of goodness-of-fit (GOF) plots and acceptable relative standard errors (RSEs) < 20% [66].

#### 2.6.1. Covariate Model

Following the development of an appropriate base structural model, the influence of covariates on PK parameters was evaluated using two different approaches: (i) a manual covariate model-building strategy, and (ii) an automatic covariate model-building method using the COSSAC algorithm [67] in Monolix. Age, BSA, height, and body weight were evaluated as continuous covariates, whereas gender, ethnicity, health status, and OME coadministration were considered categorical covariates.

The manual approach consisted of statistical screening followed by stepwise integration of covariates into the popPK model. Initially, correlations between covariates and PK parameters were explored based on the preliminary NCA conducted in PKAnalix. Covariates showing potential associations were further evaluated using Spearman’s correlation tests (as variables did not follow a normal distribution) for continuous covariates and analysis of variance (ANOVA) for categorical covariates. These analyses were performed using IBM SPSS Statistics (Version 29.0.2.0). Covariate-parameter relationships with statistical significance (*p* < 0.05) were selected for inclusion in the covariate submodel. Multicollinearity among preselected covariates was assessed using the variance inflation factor (VIF), and covariates with VIF > 10 were reconsidered. In cases of multicollinearity, the covariate with the less statistically significant *p*-value was excluded. Subsequently, a linear regression analysis using a stepwise procedure (combining forward selection and backward elimination) was performed to determine the optimal covariate submodel. In Monolix, covariates identified in the statistical submodel were incorporated sequentially into the structural model. The order of inclusion was guided by clinical relevance and by the magnitude of improvement in the objective function value (OFV), prioritizing covariates associated with the largest decrease (smallest *p*-value). Covariate inclusion in the full model followed by the criterion of a reduction in −2 log-likelihood (−2LL) greater than 3.85 (*p* < 0.05). The process was repeated until no further model improvement was observed. Backward elimination was then performed, whereby covariates were removed individually if the increase in −2LL was less than 10.83 (*p* < 0.001).

The automatic approach was based on the (Conditional Sampling for Stepwise Approach Based on Correlation Testes) algorithm [67] implemented in Monolix. Forward and backward stepwise procedures were subsequently applied to select covariates, as described previously. Acceptance or rejection of each covariate relationship was again based on improvements in −2LL and BICc criteria. If these criteria were not improved, the model was not retained.

#### 2.6.2. Predictive Performance Assessment of the Model

The final model was selected and evaluated based on the following criteria: GOF plots, including observations versus individual and population predictions, individual and population weighted residuals (IWRES and PWRES) versus individual predictions and time, and visual predictive check (VPC) using a 90% prediction interval. Model adequacy was further supported by reductions in the OFV and BIC, as well as low RSE of the estimated PK parameters [66].

## 3. Results

### 3.1. PBPK Model Development and Validation of PROP and OME

**Figure 1:**
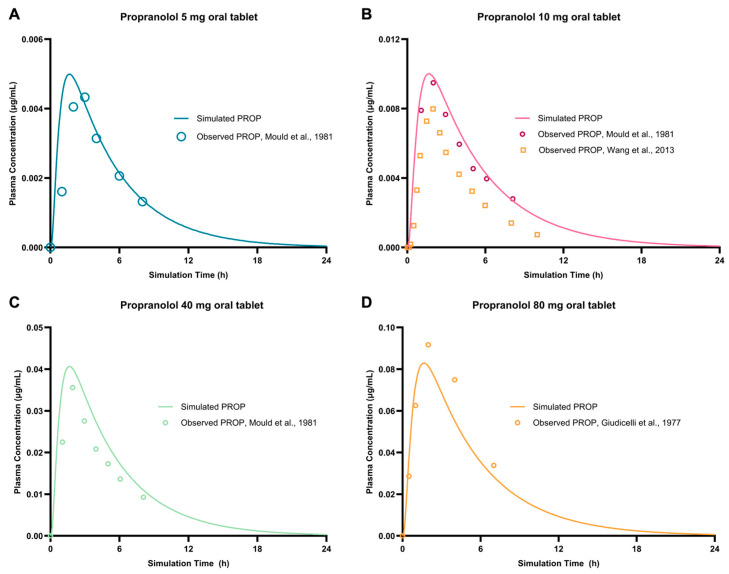
Simulated (lines) and observed (dots) plasma concentration-time profiles of (**A**) PROP following a single oral dose of 5 mg, (**B**) PROP following a single oral dose of 10 mg, (**C**) PROP following a single oral dose of 40 mg, and (**D**) PROP following a single oral dose of 80 mg in a healthy 30-year-old American man [53,54,55].

**Figure 2:**
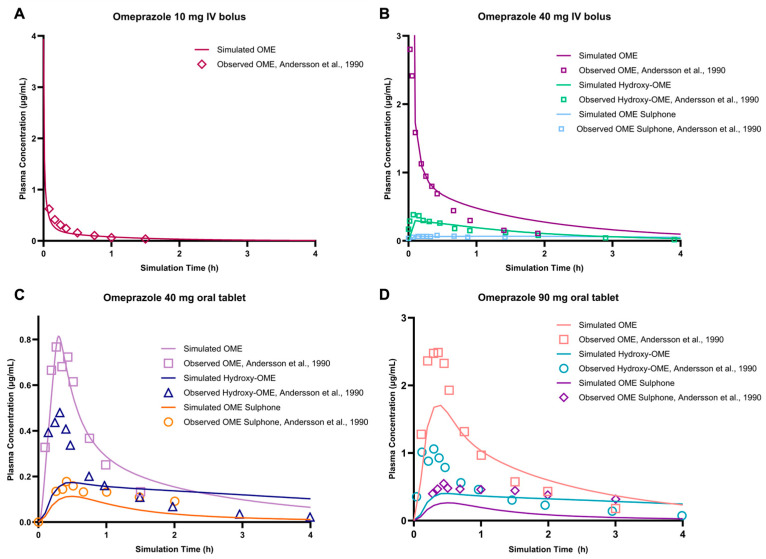
Simulated (lines) and observed (dots) plasma concentration-time profiles of (**A**) OME following a single IV dose of 10 mg, (**B**) OME following a single IV dose of 40 mg, (**C**) OME following a single oral dose of 40 mg, and (**D**) OME following a single oral dose of 90 mg in a healthy 30-year-old American man [52].

Both models show satisfactory accuracy in predicting PROP and OME PK parameters. The parameters evaluated included the C_max_, T_max_, and AUC_0−t_. The simulated concentration-time curves closely matched the observed values (Figure 1 and Figure 2). The parameters evaluated include the C_max_, T_max_, and AUC_0−t_. Similarly to the other parameters, the predicted C_max_, T_max_, and AUC_0−t_ were consistent (within a <2-FE) with reference data (observed values), as presented in Table 6.

For the PROP PBPK model, most predicted PK parameters were in good agreement with observed values across the evaluated oral doses. The predicted C_max_ values were generally consistent, with FE values ranging from 1.11 to 1.20. However, for the 10 mg dose, the model underestimated C_max_, resulting in a higher FE value of 3.66. The predicted T_max_ values were well aligned with the observed values, with FE ranging from 1.00 to 1.46. Similarly, the predicted AUC values showed good agreement with the reference data. The structural parameters of the PROP PBPK model were also consistent with literature values. The calculated volume of distribution (Vd) was 7.47 L, reflecting the extensive distribution of PROP throughout body tissues. The estimated total C*l*, comprising renal excretion and metabolic C*l*, was 46.992 L/h, which is comparable to the literature-reported C*l* of 48.6 L/h. Quantitative evaluation of model performance further supported the predictive capability of the model, with AFE values of 0.70, 0.83, and 1.22 for C_max_, T_max_, and AUC_0−t_, respectively, and corresponding RMSE values of 0.0149 µg/mL, 0.52 h, and 0.0636 µg.h/mL, all of which were within the predefined two-fold acceptance criterion.

For the OME PBPK model, predicted PK parameters following oral administration were generally consistent with observed values. At the higher oral dose of 90 mg, the predicted C_max_ and T_max_ values remained within the acceptable prediction range, with FE values of 1.66 and 1.06, respectively. However, the predicted AUC was higher than the observed value, resulting in an FE of 4.12, indicating an overestimation of systemic exposure at this level. This deviation, together with the underestimation of C_max_ observed in the OME model, highlights potential limitations in model performance and suggests that predictions outside the validated conditions for each compound should be interpreted with caution.

### 3.2. DDI Prediction

**Figure 3:**
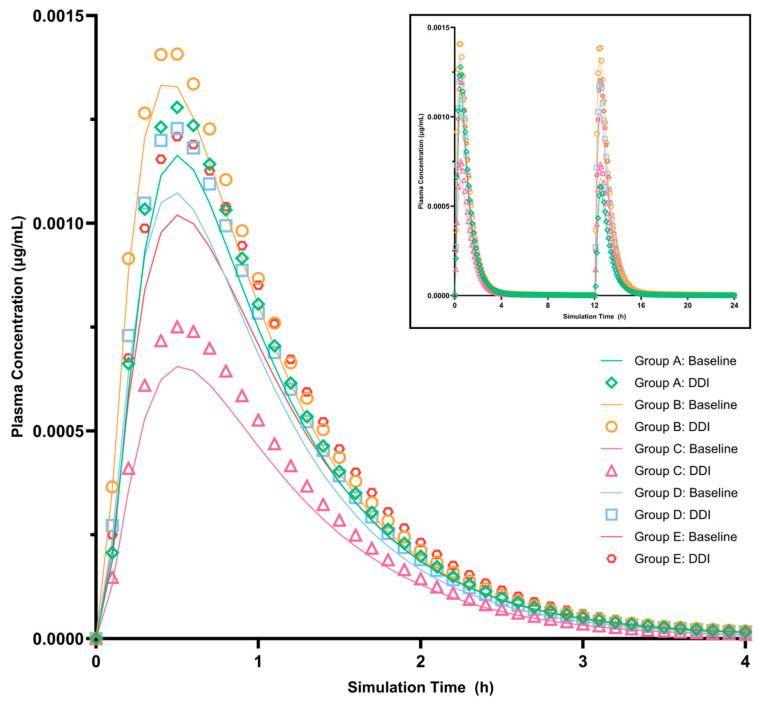
Predicted plasma concentration-time profiles in the DDI study across the different virtual population groups. Solid lines represent the predicted PROP profiles under monotherapy (baseline), while symbols represent the predicted profiles following coadministration with OME (DDI).

To evaluate the inhibitory effect of OME on PROP PK, steady-state simulations were performed using the previously validated PBPK models following coadministration of multiple PROP and OME dosing regimens. These results are detailed in the Supplementary Material (Table S3). All tested combinations of PROP and OME therapeutic regimens were classified as no interaction. Nevertheless, the absence of interaction under steady-state conditions does not preclude the occurrence of clinically relevant interactions in specific scenarios. As OME acts as the perpetrator drug, being a CYP2C19 inhibitor and a weak to moderate CYP3A4 inhibitor, and as these enzymes contribute to the metabolism of PROP, the most sensitive scenario to analyze was one that maximizes hepatic exposure to OME, temporally overlaps with the absorption of PROP, and reduces overall metabolic capacity. For these reasons, dynamic simulations were conducted in the virtual population receiving PROP 40 mg q12h in combination with OME 60 mg OD to maximize potential interaction risk by aligning peak OME exposure with PROP absorption and thereby representing conditions of highest possible metabolic competition and reduced effective enzymatic capacity for PROP clearance. Table 7 provides insights into the impact of DDI on key PK parameters, including bioavailability (F, %), C_max_, T_max_, and AUC_0−t_, collected for each virtual population group. Figure 3 illustrates the detailed PK profiles, highlighting the effect of OME coadministration on PROP plasma concentrations.

### 3.3. PopPK Demographics

A total of 125 virtual participants generated through PBPK modeling contributed 30,250 PROP plasma concentration measurements to the population analysis. No observations were excluded from the popPK model. The median age was 66 years, 56.8% of the participants were male, and 80% were American. The mean body weight was 68.7 kg, the mean height was 167.9 cm, and the mean BSA was 1.77 m^2^ (Table S4). This study included 40 healthy subjects without any associated comorbidities, 10 obese subjects, 25 with mild renal impairment, 25 with moderate renal impairment, and 25 with severe renal impairment. Patients with hepatic impairment were not included in this analysis.

### 3.4. PopPK Analysis

#### 3.4.1. Base Model and Full Covariate Analysis

The PK of PROP was best described by a two-compartment structural model with first-order absorption and linear elimination (BICc: −719,596.74; −2LL: −719,693.05). The model was parametrized in terms of the absorption rate constant (ka), with no delay in absorption onset, apparent clearance (C*l*/F), apparent intercompartmental clearance (Q/F), and apparent volumes of distribution for the central (V1) and peripheral (V2) compartments. All parameters were estimated with good precision (RSE < 8%) except for V1, which showed a higher uncertainty (RSE = 32.8%).

#### 3.4.2. Final Model

The manual covariate modeling approach identified potential effects of ethnicity on C*l* and V1 and of age on Q. However, no significant improvement in the structural model was observed following their inclusion (Table S2), and therefore none of these covariates were retained in the final model. In contrast, the COSSAC algorithm identified health status as a significant covariate on C*l* and BSA as a significant covariate on V1. Incorporation of these covariates resulted in a marked improvement in model fit (BICc: −753,391.53; −2LL: −753,549.95). Parameter estimates of the final model are detailed in Table 8. RSEs were below 30% for fixed effects, except for V1, and below 50% for random effects (IIV).

**Figure 4:**
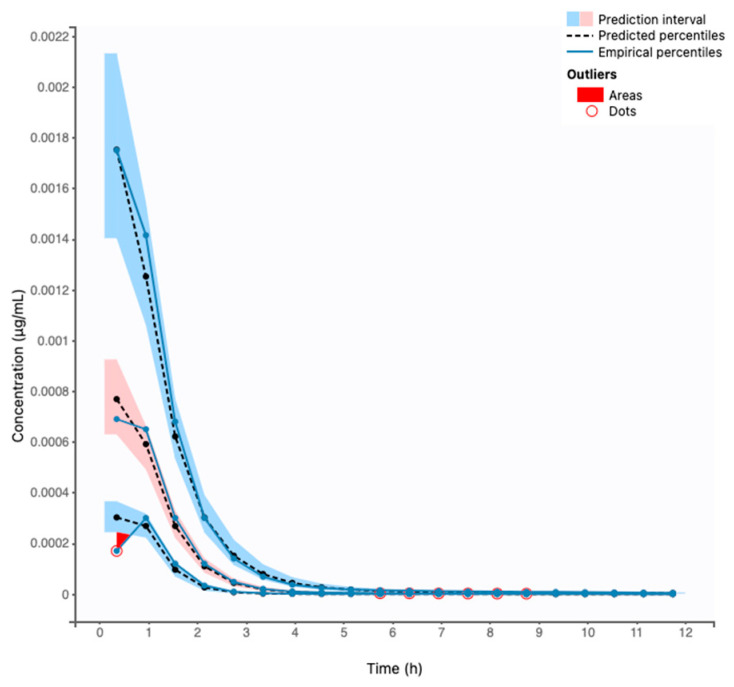
VPC for the two-compartment model with first-order absorption and linear elimination. Shaded regions represent the model-predicted 10th, 50th (median), and 90th percentile prediction intervals (displayed from bottom to top). Observed concentrations falling outside the 90% prediction interval are depicted as red points and highlighted areas.

**Figure 5:**
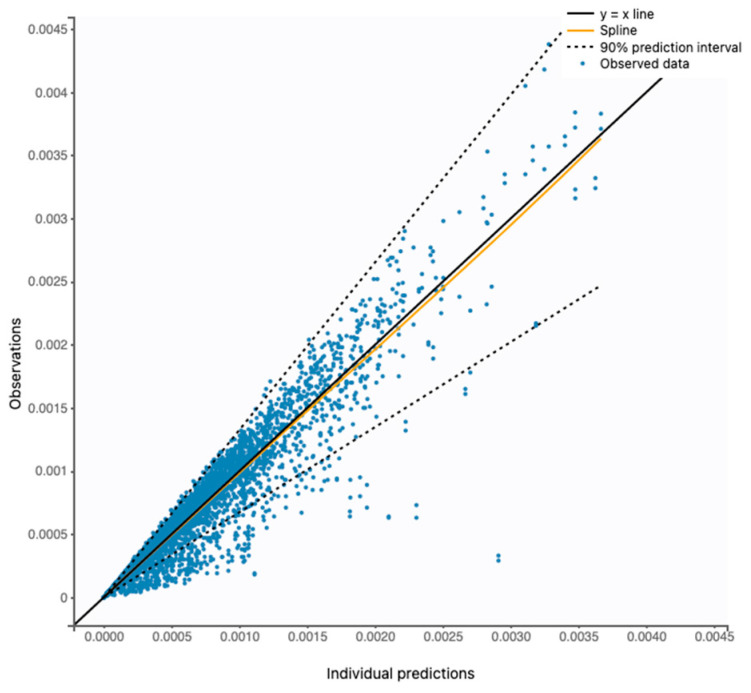
Observed PROP concentration (μg/mL) versus individual predictions (μg/mL).

GOF plots indicated that the model adequately described the observed data. The VPC showed that most of the observed concentrations fell within the 5th and 95th percentiles of the model-predicted concentrations (Figure 4), with only a small proportion of outliers (red area). Figure 5 further demonstrates close agreement between observed and predicted PROP concentrations, with observed measurements largely falling within the 90% prediction interval. The proportion of outliers was 2.13%, indicating minimal deviation from the 90% prediction range and suggesting minimal bias and strong predictive performance of the developed model. Overall, the final model adequately captured both the central tendency and the extent of variability in the oral PROP PK profile.

### 3.5. Influence of Patient-Specific Covariates in Propranolol

Our popPK analysis identified a significant association between health status and C*l*, as well as a significant effect of BSA on V1. Table 9 presents the numerical values of C*l* across the different subgroups stratified by health status, and values of V1 across three BSA categories (low, moderate, and high BSA). Geometric means and corresponding standard deviations were derived from the compartmental analysis.

**Figure 6:**
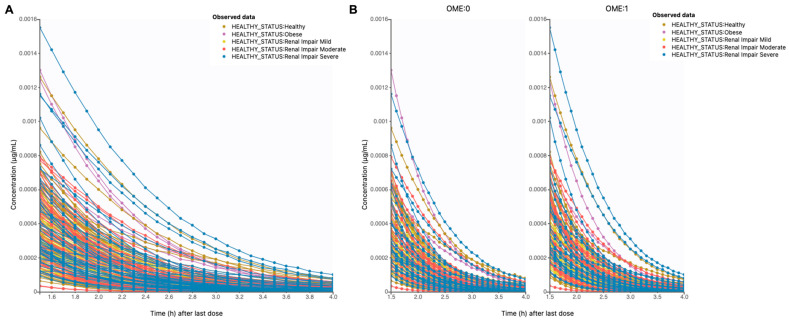
Effect of health status on C*l*. Elimination phase of the PROP PK profile in the virtual population stratified into five health status categories: (i) healthy, (ii) obese, (iii) mild renal impairment, (iv) moderate renal impairment, and (v) severe renal impairment. Panel (**A**) illustrates plasma concentration-time data within the virtual cohort, whereas panel (**B**) presents the corresponding observed concentrations collected at two distinct phases of the virtual clinical study: PROP administered as monotherapy (OME:0) and following coadministration with OME (OME:1).

**Figure 7:**
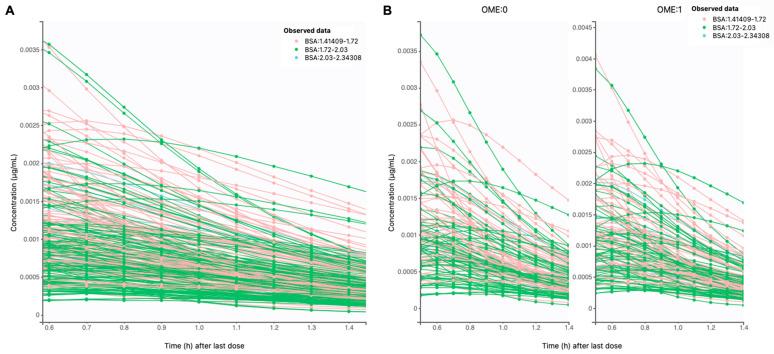
Analysis of the effect of BSA on V1. Distribution phase of the PRIP PK profile in the virtual population stratified into three BSA categories: (i) 1.41–1.72 m^2^, (ii) 1.72–2.03 m^2^, and (iii) 2.03–2.34 m^2^. Panel (**A**) presents plasma concentration-time measurements in the virtual population, while panel (**B**) exhibits the observed data from the same population at two different time points of the virtual clinical trial: intake of PROP alone (OME:0) and during concomitant administration of PROP and OME (OME:1).

A more detailed evaluation of the popPK profiles further supports these findings. Figure 6A depicts the descending phase of the PROP PK profile beginning at approximately 1.5 h, corresponding to the drug’s elimination phase, in the population stratified by health status. Figure 7A illustrates the distribution phase of the concentration-time curve, occurring approximately between 0.6 and 1.4 h, in the population stratified by BSA. In addition, to explore the impact of individual patient characteristics on the interaction between PROP and OME, the same covariate-PK parameter relationships were analyzed under PROP monotherapy (sequence I of the virtual clinical trial) and, following the washout period, during concomitant administration of PROP and OME (sequence II) (Figure 6B and Figure 7B).

## 4. Discussion

PROP remains one of the most widely prescribed β-blockers worldwide [4,5] and is indicated for a broad range of therapeutic conditions. Nevertheless, this compound is deemed clinically critical given the high incidence of reported adverse events [3]. Beyond its documented misuse and overdose, the PK of PROP is characterized by substantial variability driven by multiple determinants, including patient-specific factors, environmental influences, and DDIs [13]. These sources of variability warrant systematic investigation to identify potential risk factors associated with PROP-related toxicity. In the present study, two whole-body PBPK models capable of describing the oral PK of PROP and OME were successfully developed and evaluated in the target population. Model predictions were consistent with the clinical data used for model development and validation, with most of the predicted exposure metrics falling within the 2-fold error range, hence providing confidence in their suitability for predicting plasma concentration-time profiles in a virtual population.

Subsequently, the DDI between PROP and OME was explored across 16 scenarios, combining four therapeutic regimens of each drug at steady state. No clinically significant interaction was predicted under these conditions, which was consistent with findings reported by Henry et al. [27]. In their study, co-administration of OME 20 mg OD, the most commonly prescribed dose for peptic ulcer disease, did not significantly alter the steady-state kinetics of PROP. Although a slight reduction in PROP clearance was observed, it did not reach statistical significance within the study design. Nonetheless, OME is a potent inhibitor of certain metabolic pathways in humans. As an inhibitor of CYP2C19 and a moderate inhibitor of CYP3A4, enzymes involved in PROP metabolism, a mechanistic basis for interaction exists. Therefore, further exploration of the DDI across clinically plausible dosing scenarios was warranted. Accordingly, we performed a dynamic simulation in our virtual population receiving PROP 40 mg q12h in combination with OME 60 mg OD, enabling assessment of potential interaction under conditions expected to enhance metabolic inhibition. Our results show that the systemic exposure to PROP tends to increase in the presence of OME. The ratio, corresponding to the comparison between values in the presence of the inhibitor (OME) and baseline values (without inhibitor), for F closely mirrored the ratio observed for AUC, with an approximate 4–20% increase in overall exposure when PROP was coadministered with OME. These findings are mechanistically plausible considering that OME inhibits CYP2C19. Inhibition of an enzyme involved in PROP metabolism reduces the C*l* of the compound, which in turn leads to increases in F, AUC, and C_max_. It is well established that this β-blocker undergoes extensive hepatic first-pass extraction, resulting in relatively low amounts of drug reaching the bloodstream [12,13]. Current recommended and prescribed doses of PROP take this limited bioavailability into account; thus, any condition that moderately increases systemic exposure should be carefully evaluated. An 8–20% increase in C_max_ was also observed, with a more pronounced effect in patient groups with renal disease (moderate and severe renal impairment). Previous studies have reported that plasma concentrations following a single oral dose may be approximately two- to three-fold higher in patients with chronic kidney disease (CKD) compared with healthy individuals [68]. In these patients, intrinsic C*l* is generally reduced, which increases the fraction of drug reaching the systemic circulation as well as the effective absorption rate. Consequently, PK parameters such as F, C_max_, AUC, and T_max_ tend to be greater compared with healthy individuals. This evidence aligns with our findings: even baseline values were higher in patients with renal impairment. When these same patients were exposed to the coadministration of OME and PROP, the DDI ratio was approximately two-fold higher than that observed in healthy subjects. This observation suggests that the prescription of PROP in patients with renal disease may require more careful evaluation and supports the concept that IIV can modulate the magnitude of DDIs.

These findings are particularly relevant for drugs such as PROP, for which toxicity is concentration-dependent. Even moderate increases in systemic exposure may become clinically significant in vulnerable populations, including patients with impaired renal function who may already present altered physiological reserves and increased susceptibility to cardiovascular and CNS adverse effects. Several studies suggest that toxic plasma concentrations of PROP occur above 2 µg/mL [69], with life-threatening levels reported above 3 µg/mL [70]. Toxic doses reduce calcium influx and cyclic AMP levels in cardiac myocytes, leading to bradycardia and hypotension [3]. Plasma concentrations predicted in our study (Figure 3) never exceed 15 ng/mL, even under the DDI scenario, suggesting no apparent risk of toxicity under the investigated conditions.

The plasma concentration-time curves for each patient group during the first period of the virtual clinical trial (PROP monotherapy) exhibited the same overall profile as those observed during the second period, in which patients received PROP concomitantly with OME (Figure 3). This indicates that OME’s inhibitory effect does not significantly modify the structural PK behavior of PROP, but rather affects the magnitude of systemic exposure, consistent with the numerical results described above. Nevertheless, clear differences between baseline and DDI scenarios were observed, which prompted further investigation through a population-based analysis of PROP PK parameters.

Understanding the factors that influence drug exposure and response can inform dose optimization when considered alongside cumulative safety and efficacy data [71]. In the era of individualized pharmacotherapy, key patient-level variables include not only demographic characteristics such as age, sex, and body size descriptors but also clinical conditions and comorbidities (e.g., renal or hepatic function and the presence of several chronic diseases), pharmacogenetic factors, and detailed medication history including current treatments and co-medication, as well as environmental and lifestyle factors such as diet, smoking status, and alcohol consumption [23,72,73]. In simulation-based studies using virtual patients, researchers can generate databases incorporating many of these variables and evaluate their potential influence on drug PK. One of the most evident advantages of this approach is the possibility of investigating multiple simulated clinical scenarios without the ethical, financial, and logistical constraints inherent to real-world clinical trials [30]. In this study, we therefore mimicked a two-period fixed-sequence clinical trial design in which virtual patients generated through PBPK modeling first received PROP alone, followed by co-treatment with PROP and OME. This strategy enabled the identification of the DDI as a covariate effect and allowed the exploration of additional patient-level data that may influence the PK of PROP.

PROP’s PK were described using a two-compartmental structural model with first-order absorption and linear elimination. In this framework, orally administered PROP enters a GI deposition compartment and is subsequently absorbed into the systemic circulation according to first-order kinetics. Although PROP has been reported to exhibit nonlinear PK due to saturation of hepatic uptake processes and first-pass metabolism following oral administration [13], within the therapeutic dose explored in this study, the systemic disposition of PROP can be adequately described using a structural model with linear elimination. The assumption of a two-compartment distribution is also biologically and pharmacologically conceivable, given that PROP is a highly lipophilic compound and is therefore extensively distributed throughout body tissues [13,14,15,74].

Systematic covariate analysis identified health status as a statistically significant descriptor of variability in PROP PK at the level of C*l*, whereas BSA had a significant effect on drug distribution, specifically on the central compartment volume (V1). Several studies investigating IIV in the PK of PROP have been published since its initial discovery in the 1960s [1,13]. Evidence suggests that age may influence peak plasma concentrations, with higher PROP plasma levels generally observed in elderly individuals compared with young adults [75,76]. Although dose scaling down based on body weight is typically performed in pediatric populations, substantial variability in PROP exposure has still been reported among children. In the present study, pediatric patients were not included, as the concomitant use of PROP and OME is relatively uncommon in this population. In contrast, in adult populations, the likelihood of requiring both medications concurrently is considerably higher. According to 2023 prescription statistics in the United States, PROP was prescribed more than 9 million times [77], while OME accounted for over 45 million prescriptions [78]. Castleden et al. [76] investigated the effect of age on hepatic metabolism of PROP and demonstrated that elderly subjects exhibited higher plasma concentrations than younger adults following similar oral doses. The authors initially hypothesized that reduced volume of distribution or differences in absorption might explain these observations, although no differences in distribution volume were observed, and absorption was complete in study participants. A more plausible explanation is decreased elimination in the elderly, as hepatic blood flow and first-pass metabolism decline with age.

Sex-related discrepancies in PROP metabolism have also been reported [79]. Walle et al. [80] examined the influence of circulating testosterone and estradiol levels on PROP metabolic pathways, finding a positive correlation between testosterone concentrations in men and their PROP C*l* rates. Other studies have supported these results, showing that CYP-mediated side-chain oxidation of PROP was approximately 140% higher in men than in women, and glucuronidation was 50% higher in men [81]. Similarly, Xie et al. [82] reported that women exhibited slower PROP elimination, consistent with higher AUC and C_max_ values observed in their study. Overall, sex is widely recognized as a relevant factor in drug PK. However, multiple physiological differences exist between men and women. For instance, women generally have a higher body fat percentage, affecting drug distribution; lower glomerular filtration rates, affecting renal C*l*; and different CYP enzyme activities, influencing metabolism rates [83]. In our model, sex was not flagged as a statistically significant covariate, which may be explained by several points: (i) the effect of sex may be implicitly captured by BSA, as males generally have higher BSA; (ii) the magnitude of the sex-related effect may be relatively small compared with other sources of variability; (iii) the current model does not explicitly account for circulating hormone levels such as testosterone and estradiol; (iv) the use of a PBPK-generated virtual population may limit physiological differences between females and males, particularly in the parametrization of CYP enzyme activity. Nevertheless, these considerations do not undermine previously published evidence.

Another inference that can be drawn from our popPK analysis, following the DDI predictions, is that ethnicity does not appear to significantly influence PROP PK. Interestingly, the DDI magnitude observed in Japanese patients included in this virtual clinical trial was comparable to that observed in American patients, with similar baseline values (PROP as monotherapy). Although approximately 1.42 million single-nucleotide polymorphisms (SNPs) have been identified [84], and about 93% of known genes contain SNPs, suggesting that genetic variability may occur in drug-metabolizing enzymes [73], ethnicity did not appear to be a major determinant in our simulations. Dorji and colleagues [85] systemically reviewed polymorphisms in CYP2C9, CYP2C19, CYP2D6, and CYP3A5 among East and Southeast Asian populations. For CYP2C19, Asian people exhibit a higher frequency of the *2 allele [86] and also carry the *3 allele, which is exclusive to this population [86,87]. Many alleles from *2 to *35 are defective, resulting in reduced or absent enzyme activity. Japanese individuals are unlikely to be ultrarapid metabolizers (UMs), as the frequency of the *17 allele, associated with ultrarapid enzymatic activity, is very low [86,88,89]. Based on this evidence, one might expect that genetic variations would amplify the magnitude of the DDI. Further studies indicate considerable phenotypic variability within Asian populations. In fact, in our exploratory analysis of PK parameter distributions across covariates, the Asian individuals included in the study showed wide dispersion of AUC and C_max_ values, which likely explains why ethnicity was not retained as a significant covariate in our model. Regarding CYP2D6, the *10 variant is highly prevalent among Asians and is associated with reduced enzymatic activity. However, the intermediate metabolizer (IM) phenotype predominates, whereas the poor metabolizer (PM) phenotype remains relatively rare [90,91]. Since CYP2D6 is involved in PROP metabolism, substantial alterations in PROP metabolism driven by CYP2D6 genetic variation would not necessarily be expected in Japanese individuals.

In the first part of our study, we observed that renal function could intensify the DDI, and even baseline values were increased compared with those obtained in healthy individuals. Renal impairment has been associated not only with reduced drug excretion but also with alterations in absorption, renal and hepatic metabolism, plasma protein binding, and drug distribution [92,93]. Although PROP is not primarily eliminated via the kidneys, its PK may still be affected by impaired renal function. Our population results indicate that health status could be a covariate with a measurable impact on PROP PK. C*l* values for healthy individuals were estimated at 27 L/h, increasing to above 30 L/h in patients with renal impairment, with particularly elevated values observed in individuals with mild renal impairment, where C*l* reached 42 L/h. This trend can be visualized in Figure 6, where subjects with mild renal impairment present steeper declines in plasma concentrations over time, consistent with the higher C*l* estimated by the popPK model. Conversely, the profile of healthy individuals shows comparatively slight decreases in concentration, reflecting their lower estimated C*l* values. Under the coadministration scenario of PROP and OME, PROP concentration curves are generally higher than those observed under monotherapy conditions for the same groups, reinforcing the increase in systemic exposure previously described. In patients with renal impairment, this increase appears to be more pronounced than in healthy individuals, highlighting that the interaction may be amplified in this population. Nevertheless, the literature states that non-renal metabolic pathways are reduced in CKD [94,95,96,97,98], a progressive and irreversible condition characterized by the gradual loss of kidney function over time. Several factors have been proposed to affect drug-metabolizing enzymes in CKD, including the accumulation of uremic toxins, hormonal alterations, changes in gut microbiota, and systemic inflammation. Multiple in vivo and in vitro studies using rat models of both chronic and acute renal failure have demonstrated a downregulation of CYP enzyme activity [92]. In addition, one of the metabolic pathways of PROP involves glucuronidation. The metabolites formed through this reaction are highly polar and are efficiently excreted via renal mechanisms such as tubular secretion. In patients with renal dysfunction, these glucuronide conjugates may accumulate in the bloodstream, potentially leading to toxic effects [96].

Ultimately, our model identified a significant correlation between BSA and V1. For patients classified in the low-BSA group, the estimated V1 was 1.68 L, increasing progressively with higher BSA values. In contrast, individuals in the high-BSA group presented a markedly higher V1, reaching 19 L. This relationship can also be visually confirmed in Figure 7, where individuals with higher BSA exhibit lower plasma concentrations, while those with lower BSA show higher concentrations, reflecting the rise in V1 as BSA increases. These results are not unexpected, as BSA is associated with several physiological factors, including blood volume, body mass, organ size, and blood flow, all of which can influence drug distribution. Furthermore, the physicochemical properties of PROP may also help explain these observations. Although a high BSA does not necessarily imply obesity, obese individuals typically present BSA values between 2.0 and 2.2 m^2^, mainly due to increased body weight and height. Therefore, it is reasonable to assume that some of the individuals with the highest BSA values included in this study may correspond to obese subjects. A recent study conducted by Mortlock et al. [99] investigated the PK of PROP in individuals with healthy weight and obesity. Given that PROP is a lipophilic drug, an increased volume of distribution in obese patients would generally be expected. However, most studies analyzed by Mortlock et al. reported a decrease in both volume of distribution and C*l* in obese cohorts compared with individuals with normal body weight. Several hypotheses have been proposed to explain this observation, including differences in tissue blood flow, plasma protein binding, and hepatic C*l*, although none fully explain why PROP does not consistently follow the typical pattern observed for lipophilic drugs. In general, lipophilic compounds tend to exhibit greater affinity for nonpolar environments, facilitating tissue penetration and leading to a larger volume of distribution [100]. Interestingly, our results follow this expected PK behavior, despite the contrasting draws reported by Mortlock et al. [99].

To the best of the author’s knowledge, only two studies have investigated sources of PROP PK variability using mechanistic modeling approaches. Kalam et al. [1] developed a PBPK model to characterize differences in PROP PK between healthy subjects and patients with liver cirrhosis. They reported a significant increase in plasma PROP concentrations and a reduction in C*l* with progressive stages of cirrhosis. Based on these findings, the authors proposed model-informed dose adjustments for PROP in cirrhotic patients: reducing the oral dose from 8 mg to 6 mg, 4 mg to 2 mg, and 2 mg to 1.5 mg for patients with mild, moderate, and severe cirrhosis, respectively. The second study by Lee et al. [101] evaluated the impact of CYP2D6 genotypes on PROP PK in Korean individuals using PBPK modeling. Their results indicated that the CYP2D6*10/*10 genotype, corresponding to an IM/PM phenotype, significantly increased PROP exposure compared with wild-type subjects. Our work represents the third study addressing IIV in PROP PK, introducing a novel factor: the coadministration of a drug with potential CYP-mediated interactions, using the two most commonly applied pharmacometrics approaches, PBPK and popPK. Nevertheless, several limitations should be acknowledged. The observed data used to develop and validate the PBPK models were extracted from published plasma concentration-time curves using a digitalization tool, which inevitably introduces errors. Although the PBPK models were generally validated, some discrepancies between predicted and observed PK parameters were spotted, meaning that the simulations inherently carry a degree of inaccuracy. Another limitation relates to the use of a virtual population, which may not fully capture the complexity of physiological variability present in real patients. Additional potentially relevant covariates, such as hormonal levels, genetic background, a wider age range, and comorbidities affecting drug PK, were not explicitly incorporated into the model. Furthermore, this study relied on simulated clinical scenarios rather than prospective or retrospective clinical data, which may limit the direct translation of the findings into clinical practice. These results are dependent on the assumptions embedded within the virtual clinical trial framework; thus they should not be interpreted as confirmatory clinical evidence. Accordingly, the present findings are best positioned as hypothesis-generating, providing a structured basis to inform future study design, support the identification of relevant covariates, and prioritize scenarios for subsequent clinical evaluation. Finally, the analysis focused on a single therapeutic regimen, and therefore, the conclusions may not necessarily extend to other dosing regimens or chronic treatment settings.

### Implications in Clinical Care

Considering the widespread use of PROP in the management of CVD and the relatively high incidence of toxicity cases, it is crucial to comprehensively investigate the sources of PK variability to generate knowledge that can improve the drug’s efficacy and safety, while minimizing the occurrence of adverse effects such as bradycardia, hypotension, fatigue, drowsiness, sleep disturbances, depression, bronchospasm, impotence, and masking of hypoglycemic symptoms [6].

The PK of PROP is notoriously variable, and our results denote that renal dysfunction and BSA are two patient characteristics that should be prudently considered during drug prescription, suggesting that standard fixed dosing may not be optimal for certain patient populations. However, the toxic plasma concentrations reported for PROP are around 2 µg/mL [69], whereas our model predicted plasma concentrations of up to 4.5 ng/mL, which remain well below the established toxicity threshold. Thus, a 40 mg dose of PROP is likely to be safe despite the variability observed in the simulated population.

Furthermore, PROP and OME are commonly prescribed medications for conditions that frequently coexist in the same patient. Therefore, evaluating the potential interaction between these drugs is clinically relevant. The increased PROP exposure detected under coadministration with OME advocates that patients receiving both medications may require closer monitoring or potential dose adjustments to avoid excessive β-blockade and related adverse effects. Although the results obtained may not yet be directly translatable to clinical observations, our findings help identify key determinants of PROP PK and the factors that may intensify its interaction with OME, thereby serving as an early warning signal for potential adverse drug events.

## 5. Conclusions

This study follows the same methodological approach as our previous works [102,103], which have demonstrated the reliability of virtual data in the analysis of IIV in drug PK profiles. Here, we provide a popPK characterization of PROP and its interaction with OME, highlighting key sources of variability that may influence the absorption, distribution, metabolism, and excretion of PROP. We successfully designed a model-based virtual clinical trial, mimicking a two-period fixed-sequence clinical study, using virtual patients generated through PBPK modeling. The resulting PK and patient-level data were subsequently used for popPK analysis, representing an innovative strategy within the field of pharmacometrics.

Overall, our simulations support previous clinical observations suggesting the absence of a clinically relevant PROP-OME interaction at therapeutic doses. Nevertheless, the identification of renal impairment stage and BSA as potential factors associated with PROP exposure may contribute to improving individualized therapeutic decision-making. In addition, although no clinically relevant DDI was identified between PROP and OME, the increased PROP exposure observed during coadministration suggests that some patients may benefit from closer monitoring during combined therapy. Future studies integrating clinical PK data and more diverse patient populations, potentially including data derived from patient-specific organoids, could further expand and refine this framework. In particular, the integration of VT strategies with PBPK modeling represents a promising next step toward fully individualized simulation platforms. Ultimately, such efforts may contribute to the development of MIPD strategies for PROP and similar drugs.

## Figures and Tables

**Table 1:** Drug-specific and system-related input parameters used for the development of the PROP PBPK model.

Parameter	Value	Reference
Physicochemical Properties		
MW (g/mol)	259.35	ADMET Predictor^®^, [1]
Water Solubility (mg/mL) @ pH 10.11	2.63	ADMET Predictor^®^
pKa (base)	9.45	[1]
logP	2.89	ADMET Predictor^®^
Absorption		
P_eff_ (cm/s × 10^4^)	2.91	ADMET Predictor^®^
Diff. Coeff. (cm^2^/s × 10^5^)	0.77	GastroPlus^®^ Default
Particle density (g/mL)	1.2	GastroPlus^®^ Default
Mean precipitation time (s)	900	GastroPlus^®^ Default
Particle size (μm)	25	GastroPlus^®^ Default
Absorption model ASF (cm^−1^)	OptlogD model SA/V 6.1, used to scale passive effective permeability across different intestinal regions, adjusting for variations in the surface/volume ratio and pH along the GI tract	
Distribution		
F_up_ (%)	10	[1]
B:P	0.89	[1]
Partition Coefficient Model		Poulin and Theil Extracellular method [39]
Tissues		Perfusion-limited rate
Metabolism		
CL_H_ (L/h)	46	[1]
CYP1A2 K_m_ (mg/L) (PBPK)	0.38	ADMET Predictor^®^
CYP1A2 V_max_ (mg/s/mg enzyme) (PBPK)	6.12 × 10^−4^	ADMET Predictor^®^
CYP2C19 K_m_ (mg/L) (PBPK)	12.97	ADMET Predictor^®^
CYP2C19 V_max_ (mg/s/mg enzyme) (PBPK)	0.016	ADMET Predictor^®^
CYP2D6 K_m_ (mg/L) (PBPK)	0.33	ADMET Predictor^®^
CYP2C19 V_max_ (mg/s/mg enzyme) (PBPK)	1.52 × 10^−3^	ADMET Predictor^®^
Excretion		
CL_R_ (L/h)	0.975	[1]

MW, molecular weight; pKa, ionization constant; logP, octanol/water partition coefficient; P_eff_, effective human jejunal permeability; Diff. Coeff., differential coefficient; F_up_, fraction unbound in plasma; B:P, blood/plasma ratio; CL_H_, hepatic clearance; CYP, cytochrome P450; K_m_, Michaelis-Menten constant; V_max_, maximum velocity; CL_R_, renal clearance.

**Table 2:** Input parameters used for the development of the OME PBPK model.

Parameter	Value	Reference
Physicochemical Properties		
MW (g/mol)	345.42	[40,45,46]
Water Solubility (mg/mL) @ pH 7.4	0.0823	[40,47]
pKa (base)		[40,48]
logP	2.23	[40,46]
Absorption		
P_eff_ (cm/s × 10^4^)	12	[40]
Diff. Coeff. (cm^2^/s × 10^5^)	0.71	GastroPlus^®^ Default
Particle density (g/mL)	1.2	GastroPlus^®^ Default
Mean precipitation time (s)	900	GastroPlus^®^ Default
Particle size (μm)	25	GastroPlus^®^ Default
Absorption model ASF (cm^−1^)	OptlogD model SA/V 6.1, used to scale passive effective permeability across different intestinal regions, adjusting for variations in the surface/volume ratio and pH along the GI tract	
Distribution		
F_up_ (%)	10.2	ADMET Predictor^®^
B:P	0.6	[40,49]
Partition Coefficient Model		Lucakova method [50]
Tissues		Perfusion-limited rate
Metabolism		
Formation of 5′-O-desmethylomeprazole		
CYP2C19 K_m_ (mg/L) (PBPK)	0.811	[40,42]
CYP2C19 V_max_ (mg/s/mg enzyme) (PBPK)	5.115 × 10^−4^	Initially informed in vitro [42], then fitted by [40]
CYP2C9 K_m_ (mg/L) (PBPK)	73.92	[40,42]
CYP2C9 V_max_ (mg/s/mg enzyme) (PBPK)	7.97 × 10^−5^	[40,42]
CYP3A4 K_m_ (mg/L) (PBPK)	181	[40,42]
CYP3A4 V_max_ (mg/s/mg enzyme) (PBPK)	3.676 × 10^−3^	Initially informed in vitro [42], then fitted by [40]
CYP3A7 K_m_ (mg/L) (PBPK)	923.1	[40]; calculated from Km for CYP3A4 [51]
CYP3A7 V_max_ (mg/s/mg enzyme) (PBPK)	9.09 × 10^−4^	[40]; calculated from Km for CYP3A4 [51]
Formation of hydroxy-OME		
CYP2C19 K_m_ (mg/L) (PBPK)	1.657	[40,52]
CYP2C19 V_max_ (mg/s/mg enzyme) (PBPK)	3.67 × 10^−4^	Initially informed in vitro [52], fit to PK data
CYP2C9 K_m_ (mg/L) (PBPK)	141.3	[40,52]
CYP2C9 V_max_ (mg/s/mg enzyme) (PBPK)	1.803 × 10^−3^	Initially informed in vitro [52], then fitted by [40]
CYP3A4 K_m_ (mg/L) (PBPK)	117.4	[40,52]
CYP3A4 V_max_ (mg/s/mg enzyme) (PBPK)	8.39 × 10^−4^	[40,52]
CYP3A7 K_m_ (mg/L) (PBPK)	598.23	[40]; calculated from Km for CYP3A4 [51]
CYP3A7 V_max_ (mg/s/mg enzyme) (PBPK)	2.1 × 10^−4^	[40]; calculated from Vmax for CYP3A4 [51]
Formation of Sulphone OME		
CYP3A4 K_m_ (mg/L) (PBPK)	28.57	[40,42]
CYP3A4 V_max_ (mg/s/mg enzyme) (PBPK)	1.5 × 10^−3^	Initially informed in vitro [42], then fitted by [40]
CYP3A4 K_m_ (mg/L) (gut)	28.57	[40,42]
CYP3A4 V_max_ (mg/s/mg enzyme) (gut)	9.21 × 10^−2^	Initially informed in vitro [42], fit to PK data
CYP3A7 K_m_ (mg/L) (PBPK)	145.7	[40]; calculated from Km for CYP3A4 [51]
CYP3A7 V_max_ (mg/s/mg enzyme) (PBPK)	3.75 × 10^−4^	[40]; calculated from Vmax for CYP3A4 [51]

MW, molecular weight; pKa, ionization constant; logP, octanol/water partition coefficient; P_eff_, effective human jejunal permeability; Diff. Coeff., differential coefficient; F_up_, fraction unbound in plasma; B:P, blood/plasma ratio; CYP, cytochrome P450; K_m_, Michaelis-Menten constant; V_max_, maximum velocity.

**Table 3:** Clinical study design and subject characteristics used for PROP and OME PBPK model development and verification.

	Participants (*n*, Sex)	Dose and Regimen	Administration Route	Age (Years, Range)	Body Weight (kg, Range)	Reference
PROP PBPK model development	14 males 4 females	5 mg SD	PO	34	NA	[53]
PROP PBPK model validation	14 males 4 females	10 mg SD	PO	34	NA	[53]
	20 males	10 mg SD	PO	18–60	60–81 *	[54]
	14 males 4 females	40 mg SD	PO	34	NA	[53]
	3 males 3 females	80 mg SD	PO	20–28	41–70	[40,55]
OME PBPK model development	10 males	10 mg SD 40 mg SD	IV	19–27	70–86	[40]
OME PBPK model validation	10 males	40 mg SD 90 mg SD	PO	19–27	70–86	[40]

* Within 15% of ideal weight; SD, single dose; IV, intravenous administration; PO, oral administration; NA, not available.

**Table 4:** Inhibition parameters of OME and metabolic profile of PROP (CL_int_ $=V_{max}/K_{m}$).

Drug	CYP450 Target	Parameter	Value	Units	Reference
OME	CYP2C19	K_i_	1.1	µM	[40,60,61]
		K_inact_	0.048	min^−1^	
	CYP3A4	K_i_	52	µM	[40,60]
		K_inact_	0.029	min^−1^	
PROP	CYP1A2	CL_int_	$1.97\times 10^{5}$	L/h	Detected from information in Enzyme Table of GastroPlus^®^
		fm	51.67	%	
	CYP2C19	CL_int_	$9.85\times 10^{3}$	L/h	
		fm	2.59	%	
	CYP2D6	CL_int_	$1.18\times 10^{4}$	L/h	
		fm	3.09	%	

K_i_, inhibition constant; K_inact_, inactivation rate constant; CL_int_, intrinsic clearance; fm, metabolized fraction.

**Table 5:** Characteristics of the virtual patient groups included in the virtual clinical trial.

Group	*n*	Ethnicity	Health Status	Age Range (Years)	BMI Range (kg/m^2^)	Weight Range (kg)
A	25	American	Healthy	18–80	15.64–35	54–115
B	25	Japanese	Healthy	18–80	15.64–35	54–115
C	25	American	Mild renal impairment	59–80	15.64–25.22	49–79
D	25	American	Moderate renal impairment	59–80	15.64–25.22	49–79
E	25	American	Severe renal impairment	59–80	15.64–25.22	49–79

**Table 6:** Observed (reference) and predicted PK parameters following single oral doses of 5, 10, 40, and 80 mg PROP simulated using the PROP PBPK model and following single IV doses of 10 and 40 mg and single oral doses of 40 and 90 mg OME simulated using the OME PBPK model.

Drug	Dose Regimen	C_max_ (µg/mL)			T_max_ (h)			AUC_0−t_ (µg.h/mL)		
		Observed Value	Predicted Value	FE	Observed Value	Predicted Value	FE	Observed Value	Predicted Value	FE
PROP	5 mg SD po	0.016	0.015	1.11	2.95	2.02	1.46	0.033	0.037	1.12
	10 mg SD po	0.037	0.010	**3.66**	1.99	1.68	1.18	0.051	0.060	1.18
	40 mg SD po	0.036	0.030	1.20	1.94	1.94	1.00	0.231	0.348	1.51
	80 mg SD po	0.071	0.082	1.16	2.00	1.68	1.19	0.450	0.499	1.11
OME	40 mg SD po	1.04	0.84	1.24	0.28	0.34	1.21	0.681	1.188	1.77
	90 mg SD po	3.25	1.95	1.66	0.32	0.34	1.06	0.681	2.806	**4.12**

C_max_, maximum concentration; T_max_, time to peak concentration; AUC, area under the concentration-time curve; FE, fold error. Bold values represent results exceeding the two-fold error threshold.

**Table 7:** Predicted PK parameters following oral administration of PROP as monotherapy (40 mg q12h) and in combination with OME (60 mg OD) across the different virtual population groups.

Patient Group	GMR of F (%)			GMR of C_max_ (µg/mL)			GMR of T_max_ (h)			GMR of AUC_0−t_ (µg.h/mL)		
	Baseline	DDI	DDI Ratio	Baseline	DDI	DDI Ratio	Baseline	DDI	DDI Ratio	Baseline	DDI	DDI Ratio
A	0.112	0.122	1.084	0.00101	0.00111	1.099	0.538	0.528	0.981	1.832	1.985	1.084
B	0.217	0.233	1.038	0.00127	0.00138	1.082	0.560	0.940	1.679	1.496	1.686	1.082
C	0.117	0.131	1.121	0.00060	0.00068	1.137	0.560	0.896	1.600	1.602	1.796	1.121
D	0.161	0.184	1.142	0.00096	0.00111	1.154	0.550	1.104	2.007	2.341	2.675	1.143
E	0.165	0.198	1.195	0.00088	0.00107	1.202	0.570	1.919	3.367	2.272	2.711	1.193

GMR, geometric mean ratio.

**Table 8:** Final popPK parameter estimates.

Parameter	Estimate	RSE (%)
Fixed effects		
ka (h^−1^)	2.17	4.85
V1 (L)	12.07	42.3
Q (L/h)	1.07	11.7
V2 (L)	9.27	19.2
C*l* (L/h)	28.93	6.45
Random effects		
IIV (ka)	0.34	12.1
IIV (V1)	0.62	8.70
IIV (Q)	1.29	6.43
IIV (V2)	2.06	6.44
IIV (C*l*)	0.49	6.37

RSE, relative standard error; ka, absorption rate constant; V1, volume of distribution of the central compartment; Q, intercompartmental clearance; V2, volume of distribution of the peripheral compartment; C*l*, clearance; IIV, interindividual variability.

**Table 9:** PK parameters of PROP across the different virtual population subgroups stratified by health status and BSA. Geometric means and corresponding standard deviations (SD) are derived from compartmental analysis.

Parameter	Covariate		Geometric Mean	SD
C*l*	Health Status	Healthy	27.06	1.51
		Obese	33.91	1.59
		Renal Impairment Mild	42.03	1.51
		Renal Impairment Moderate	30.99	1.55
		Renal Impairment Severe	31.20	1.67
V1	BSA	1.41–1.72 m^2^	1.688	409.50
		1.72–2.03 m^2^	3.69	265.12
		2.03–2.34 m^2^	19.68	1.48

## Data Availability

The original contributions presented in this study are included in the article/Supplementary Material. Further inquiries can be directed to the corresponding authors.

## Supplementary Materials

The following supporting information can be downloaded at: https://www.mdpi.com/article/10.3390/pharmaceutics18060636/s1, Table S1: Drug-specific and system-related input parameters used for the development of the metabolites hydroxy-OME and OME sulphone PBPK models. Table S2: PopPK models of PROP. Table S3: Simulated impact of OME on PROP PK steady-state for multiple clinically relevant PROP and OME dosing regimens. Table S4: Demographic and clinical characteristics of patients (mean or median ± standard deviation, SD, or interquartile range, IR).
