# Supplementary material for: Model-Based Virtual Clinical Trial Reveals Renal Impairment and Body Size as Key Determinants of Pharmacokinetic Variability and Drug-Drug Interaction Risk in Propranolol Therapy
Source: Pharmaceutics. 2026 May 22;18(6):636. doi: 10.3390/pharmaceutics18060636 (PMC13306096; doi:10.3390/pharmaceutics18060636)
Supplement: Supplementary file 1 [file pharmaceutics-18-00636-s001.zip › pharmaceutics-4265647-supplementary.pdf]

Supplementary Materials: Model-based Virtual Clinical Trial Reveals Renal Impairment and Body Size as Key Determinants of Pharmacokinetic Variability and Drug-Drug Interaction Risk in Propranolol Therapy

Lara Marques [1,2] and Nuno Vale [1,2,3,*]

[1] PerMed Research Group, RISE-Health, Faculty of Medicine, University of Porto, Alameda Professor Hernâni Monteiro, 4200-319 Porto, Portugal; lara.marques2010@hotmail.com
2 RISE-Health, Department of Community Medicine, Health Information and Decision (MEDCIDS), Faculty of Medicine, University of Porto, Rua Doutor Plácido da Costa, 4200-450 Porto, Portugal
3 Laboratory of Personalized Medicine, Department of Community Medicine, Health Information and Decision (MEDCIDS), Faculty of Medicine, University of Porto, Rua Doutor Plácido da Costa, 4200-450 Porto, Portugal
* Correspondence: nunovale@med.up.pt; Tel.: +351-220426537

1. PBPK Model Development of Omeprazole

Table S1. Drug-specific and system-related input parameters used for the development of the metabolites hydroxy-OME and OME sulphone PBPK models.

Parameter	Value	Reference
**Hydroxy-OME formation**		
Water Solubility (mg/mL) @ pH 7.63	1.15	ADMET Predictor®
pKa	11.19 (acid)<br>4.23 (base)<br>2.55 (base)	ADMET Predictor®
LogP	0.86	ADMET Predictor®
P_{eff} (cm/s × 10^4)	1.02	ADMET Predictor®
Diff. Coeff. (cm²/s × 10^5)	0.7	ADMET Predictor®
Particle density (g/mL)	1.2	GastroPlus® Default
Mean precipitation time (s)	900	GastroPlus® Default
Particle size (μm)	25	GastroPlus® Default
Solubility Factor	66.07	ADMET Predictor®
FaSSIF solubility (mg/mL) @ pH 6.5	0.41	ADMET Predictor®
FeSSIF solubility (mg/mL) @ pH 5.0	0.54	ADMET Predictor®
F_{up} (%)	23.3	ADMET Predictor®
B:P	0.6	ADMET Predictor®
Partition Coefficient Model		Poulin and Theil Extracellular method
Tissues		Perfusion-limited rate
**Degradation of Hydroxy-OME**		
Lumped MP K_m (mg/L) (PBPK)	0.16	ADMET Predictor®
Lumped MP V_{max} (mg/s/mg enzyme) (PBPK)	7×10^{-7}	ADMET Predictor®
CYP3A4 K_m (mg/L) (PBPK)	24.91	ADMET Predictor®
CYP3A4 V_{max} (mg/s/mg enzyme) (PBPK)	9.47×10^{-7}	ADMET Predictor®
CYP3A7 K_m (mg/L) (PBPK)	123.44	ADMET Predictor®

CYP3A7 V_{max} (mg/s/mg enzyme) (PBPK)	2.37×10^{-7}	ADMET Predictor®
**OME sulphone formation**		
Water Solubility (mg/mL) @ pH 3.15	3.09	ADMET Predictor®
pKa	10.89 (acid)<br>3.93 (base)<br>1.79 (base)	ADMET Predictor®
LogP	2.24	ADMET Predictor®
P_{eff} (cm/s × 10^4)	0.67	ADMET Predictor®
Diff. Coeff. (cm²/s × 10^5)	0.69	ADMET Predictor®
Particle density (g/mL)	1.2	GastroPlus® Default
Mean precipitation time (s)	900	GastroPlus® Default
Particle size (μm)	25	GastroPlus® Default
Solubility Factor	449.9	ADMET Predictor®
FaSSIF solubility (mg/mL) @ pH 6.5	0.043	ADMET Predictor®
FeSSIF solubility (mg/mL) @ pH 5.0	0.15	ADMET Predictor®
F_{up} (%)	11.3	ADMET Predictor®
B:P	0.6	ADMET Predictor®
Partition Coefficient Model		Poulin and Theil Extracellular method
Tissues		Perfusion-limited rate
**Degradation of OME sulphone**		
CYP2C19 K_m (mg/L) (PBPK)	0.058	ADMET Predictor®
CYP2C19 V_{max} (mg/s/mg enzyme) (PBPK)	5.17×10^{-5}	ADMET Predictor®
CYP3A4 K_m (mg/L) (PBPK)	8.46	ADMET Predictor®
CYP3A4V_{max} (mg/s/mg enzyme) (PBPK)	9.89×10^{-4}	ADMET Predictor®
CYP3A7 K_m (mg/L) (PBPK)	42.966	ADMET Predictor®
CYP3A7 V_{max} (mg/s/mg enzyme) (PBPK)	2.47×10^{-4}	ADMET Predictor®

pKa, ionization constant; logP, octanol/water partition coefficient; P_{eff}, effective human jejunal permeability; Diff. Coeff., differential coefficient; FaSSIF, fasted state simulated intestinal fluid; FeSSIF: fed state simulated intestinal fluid; F_{up}, fraction unbound in plasma; B:P, blood/plasma ratio; CYP, cytochrome P450; K_m, Michaelis-Menten constant; V_{max}, maximum velocity.

2. PopPK Model Development

Table S2. PopPK models of PROP.

Project Name	Model Description	Error Model	–2 LL	BICc
PK_P_01	One compartment model with first-order absorption, no delay, linear elimination, and clearance as parametrization	Combined 1	–636822.84	–636756.81
PL_P_02	One compartment model with first-order absorption, lag time, linear elimination, and clearance as parametrization	Combined 1	–601702.03	–601620.87
PK_P_03	One compartment model with first-order absorption, transit compartments, linear elimination, and clearance as parametrization	Combined 1	–539716.87	–539620.57
PK_P_04	One compartment model with zero-order absorption, no delay,	Combined 1	–620446.95	–620380.92

	linear elimination, and clearance as parametrization			
PK_P_05	Two compartment model with first-order absorption, no delay, linear elimination, and clearance as parametrization	Combined 1	–719693.05	–719596.74
PK_P_06	Two compartment model with first-order absorption, lag time, linear elimination, and clearance as parametrization	Combined 1	–597890.8	–597779.36
PK_P_07	Two compartment model with first-order absorption, transit compartments, linear elimination, and clearance as parametrization	Combined 1	–677792.67	–677666.09
PK_P_08	Two compartment model with zero-order absorption, no delay, linear elimination, and clearance as parametrization	Combined 1	–569851.5	–569755.2
PK_P_09	Three compartment model with first-order absorption, no delay, linear elimination, and clearance as parametrization	Combined 1	–682538.24	–682411.66
PK_P_10	Two compartment model with first-order absorption, no delay, Michaelis Menten elimination, and clearance as parametrization	Combined 1	–697158.46	–697047.02
PK_P_11	One compartment model with first-order absorption, no delay, Michaelis Menten elimination, and clearance as parametrization	Combined 1	–620595.76	–620514.59
PK_P_12	Two compartment model with first-order absorption, no delay, linear elimination, and rate constant as parametrization	Combined 1	–697986.43	–697890.13
PK_P_13	Two compartment model with first-order absorption, no delay, linear elimination, and hybrid constant as parametrization	Combined 1	–624605.58	–624509.27
PK_P_14	Two compartment model with first-order absorption, no delay, linear elimination, and clearance as parametrization	Combined 2	–503787.42	–503691.12
PK_P_15	Two compartment model with first-order absorption, no delay, linear elimination, and clearance as parametrization	Proportional	–742259.46	–742173.47
PK_P_16	Two compartment model with first-order absorption, no delay, linear elimination, and clearance as parametrization	Constant	–503824.28	–503738.28

PK_P_17	Two compartment model with first-order absorption, no delay, linear elimination, and clearance as parametrization with ethnicity on C*l*	Proportional	–739371.55	–739280.72
PK_P_18	Two compartment model with first-order absorption, no delay, linear elimination, and clearance as parametrization with ethnicity on V1	Proportional	–736245.02	–736154.2
PK_P_19	Two compartment model with first-order absorption, no delay, linear elimination, and clearance as parametrization with age on Q	Proportional	–736587.46	–736496.64
PK_P_20	Two compartment model with first-order absorption, no delay, linear elimination, and clearance as parametrization with health status on C*l* and BSA on V1 (COSSAC algorithm)	Proportional	–753549.95	–753391.53

–2LL, –2 × log likelihood, BICc, Corrected Bayesian Information Criterion. PK_P_05 was the best structural model according to validation metrics. PK_P_15 was adjusted to achieve the best error model. PK_P_20 is the final model.

3. DDI Prediction: Steady-state Simulations

Table S3. Simulated impact of OME on PROP PK steady-state for multiple clinically relevant PROP and OME dosing regimens.

PROP Dose Regimen	OME Dose Regimen	AUC Ratio	Perpetrator Classification
PROP 40 mg q12h	OME 20 mg OD	1.002	No interaction
	OME 40 mg OD	1.003	No interaction
	OME 40 mg q12h	1.003	No interaction
	OME 60 mg OD	1.005	No interaction
PROP 80 mg OD	OME 20 mg OD	1.002	No interaction
	OME 40 mg OD	1.003	No interaction
	OME 40 mg q12h	1.003	No interaction
	OME 60 mg OD	1.005	No interaction
PROP 10 mg q6h	OME 20 mg OD	1.002	No interaction
	OME 40 mg OD	1.003	No interaction
	OME 40 mg q12h	1.003	No interaction
	OME 60 mg OD	1.005	No interaction
PROP 160 mg OD	OME 20 mg OD	1.002	No interaction
	OME 40 mg OD	1.003	No interaction
	OME 40 mg q12h	1.003	No interaction
	OME 60 mg OD	1.005	No interaction

AUC, area under the curve; q12h, every 12 hours; OD, once daily; q6h, every 6 hours.

4. PopPK Demographics

Table S4. Demographic and clinical characteristics of patients (mean or median ± standard deviation, SD, or interquartile range, IR).

Characteristics	Total (*n* = 125)
Age (years)	66 ± 15.9
Gender (*n*, %)	
Female	54, 56.8%
Male	71, 43,2%
BSA (m^2)	1.74 ± 0.19
Weight (kg)	66.9 ± 12.76
Height (cm)	167.95 ± 8.99
Ethnicity (*n*, %)	
American	100, 80%
Asian	50, 20%
Health Status (*n*, %)	
Healthy	40, 32%
Obese	10, 8%
Mild Renal Impairment	25, 20%
Moderate Renal Impairment	25, 20%
Severe Renal Impairment	25, 20%
